# Deep learning-based synthetic brain MRI for the assessment of regional atrophy patterns in neurodegenerative diseases

**DOI:** 10.1007/s00330-025-12302-9

**Published:** 2026-02-27

**Authors:** Evamaria O. Riedel, Severin Schramm, Fabian Bongratz, Martin J. Gruber, Dominik Sepp, Karolin J. Paprottka, Claus Zimmer, Benedikt Wiestler, Dennis M. Hedderich

**Affiliations:** 1 Instiute for Neuroradiology, School of Medicine and Health, TUM Klinikum Rechts der Isar, Technical University of Munich, Munich, Germany; 2 Department of Radiology, School of Medicine and Health, TUM Klinikum Rechts der Isar, Technical University of Munich, Munich, Germany; 3 Munich Center for Machine Learning, Munich, Germany; 4 AI for Image-Guided Diagnosis and Therapy, School of Medicine and Health, Technical University of Munich, Munich, Germany

**Keywords:** Brain, Alzheimer disease, Frontotemporal lobar degeneration, Magnetic resonance imaging, Deep learning

## Abstract

**Objectives:**

Assessing regional brain atrophy on 3D-T1w imaging is crucial for evaluating neurodegenerative disorders. However, high-quality volumetric imaging is not always available. Thus, AI-based algorithms were developed to generate “synthetic” 3D-T1w sequences using various clinical sequences as input. This retrospective study aims to investigate whether regional atrophy patterns are preserved in deep learning-based synthetic 3D-T1w sequences from different inputs.

**Materials and methods:**

The study included patients with Alzheimer’s disease (AD), Frontotemporal dementia (FTD), and healthy controls (HC). Probands were scanned at 3 T, and deep learning-based synthetic 3D-T1w images were generated from various inputs (3D FLAIR, 4 mm axial FLAIR, 4 mm coronal T2) using FreeSurfer-based SynthSR. Real 3D-T1w images served as the reference standard. Brain volumetry was performed using SynthSeg+ in FreeSurfer and the AssemblyNet-AD-FTD pipeline in VolBrain.

**Results:**

Global and regional volumes differed significantly between deep learning-based synthetized sequences and the reference standard 3D T1 for all subgroups and inputs (total white matter volume AD *p* = 0.0002, FTD *p* < 0.0001, HC *p* = 0.0116; total gray matter volume for AD, FTD, and HC *p* < 0.0001), except for hippocampal volumes. This systematic error in overestimating volumes affected automated disease probability prediction in FTD for all inputs (*p* < 0.0001) and in HC for coronal T2 input (adj. *p* = 0.0054).

**Conclusion:**

Deep learning-based synthetic 3D-T1w sequences introduce systematic errors in assessing global and regional brain volumetric measures, leading to overestimated volumes in controls and patients. Resulting synthetic images should be used cautiously, especially for volumetric analyses.

**Key Points:**

***Question*** *It remains unclear whether deep learning-based synthetic 3D-T1w images from various inputs preserve regional atrophy patterns sufficiently to serve as input for automated volumetry*.

***Findings*** *Deep learning-based synthetic T1w images overestimate regional and global brain volumes in neurodegenerative diseases and controls, increasing with lower quality inputs*.

***Clinical relevance*** *Deep learning-based synthetic images should only be used with caution for volumetric evaluation of brain MRI scans. If possible, 3D scans should be used as input*.

**Graphical Abstract:**

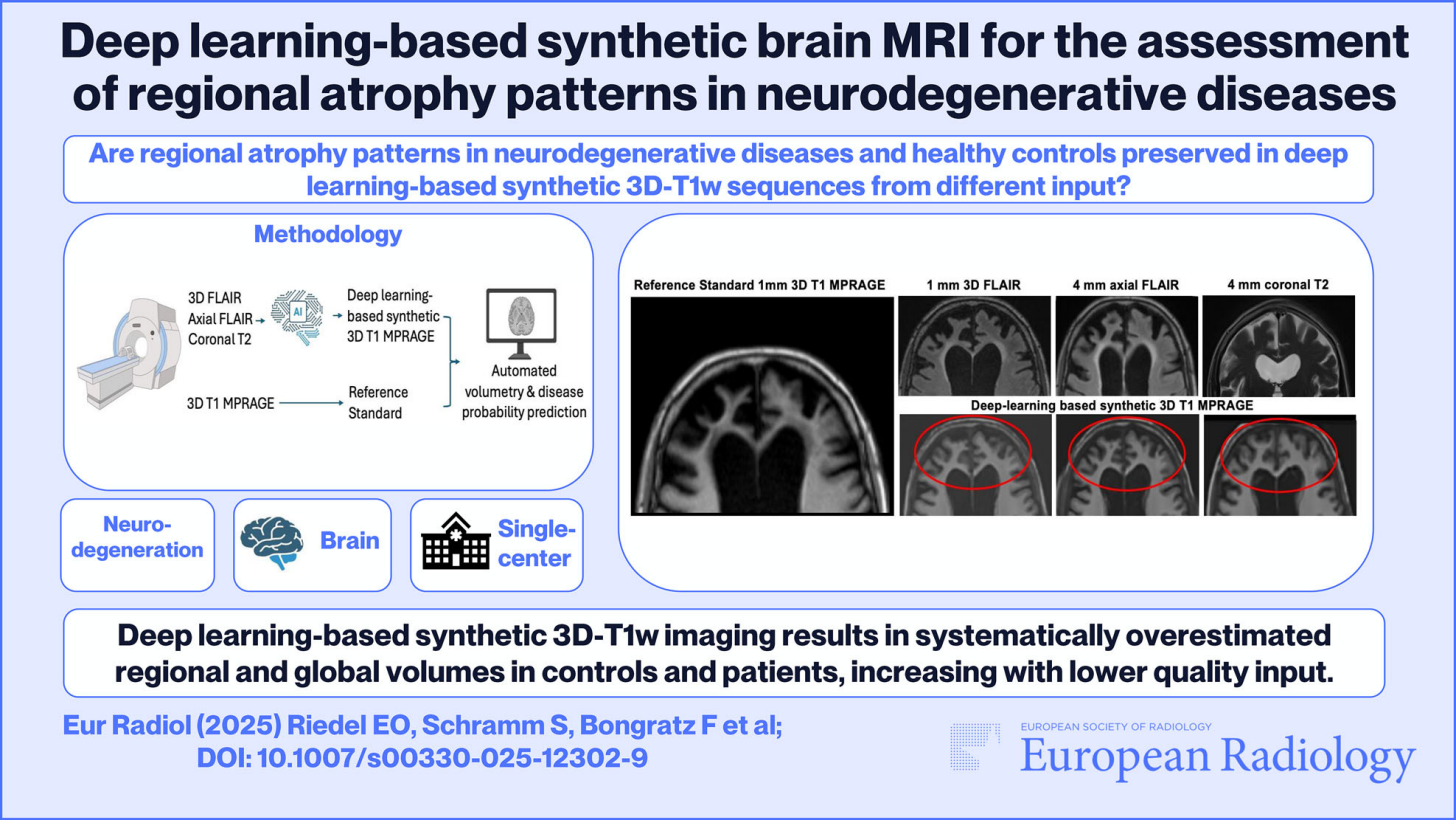

## Introduction

Neurodegenerative diseases are among the leading causes of global mortality and morbidity [1, 2]. For the following decades, a marked increase in global prevalence is predicted [2]. Brain magnetic resonance imaging (MRI) is a mainstay in the diagnostic work-up of patients with suspected neurodegenerative disorders, and particularly artificial intelligence (AI)-based methods bear great potential to improve the diagnostic decision-making by distinguishing several regional atrophy patterns. In order to assess region-specific atrophy, the reference standard for volumetric analyses is a 1-mm isotropic 3D T1 sequence (e.g., magnetization prepared—rapid gradient echo (MPRAGE)), and as such, most tools are designed to use this sequence for volumetric analysis. However, during clinical routine scans, 3D T1 is often not acquired, and a proper input sequence for automated volumetric analyses is lacking.

Generating certain synthetic sequences from different inputs based on deep learning is gaining importance in the field of radiology in general [3, 4]. High-quality isometric 3D-T1w imaging sequences can also be generated from various clinical input sequences (e.g., T1 or T2 weighted, fluid-attenuated inversion recovery (FLAIR)) using deep learning. To this end, SynthSR was released as an addition to FreeSurfer. It allows for the generation of synthetic high-resolution 3D T1-weighted brain MRIs out of multimodal input (contrast or resolution) and could demonstrate high correlations of volumes between acquired and synthetic 3D T1 sequences, as well as strong discrimination of Alzheimer’s patients using volumetry of the hippocampus [5].

However, it remains unclear whether SynthSR performs well on clinical data from patients with disease-specific, regional atrophy patterns. To bridge this gap, we investigated these deep learning-based synthetic 3D T1 sequences in AD and FTD patients based on diverse input sequences with various contrasts and resolutions. We assessed global and regional volumetric measurements of SynthSR-generated sequences compared to the reference standard, particularly focusing on regions with disease-specific atrophy. Furthermore, we analyzed whether these synthetic sequences can be used as input for an automated classification algorithm.

## Materials and methods

### Ethical approval and compliance

Institutional Review Board approval was obtained, and the requirement of written informed consent was waived by the local ethics committee due to the retrospective nature of the study (2024-71-S-CB).

### Power analysis

To assess the adequacy of the sample size, a power analysis was conducted using G*Power 3.1 for a repeated measures analysis of variance (ANOVA) (within-subjects) with four measurements, assuming a medium effect size (*f* = 0.25), α = 0.05, a correlation among repeated measures of 0.9, and a nonsphericity correction ε = 0.75, indicating that a sample size of 10 participants would achieve a statistical power of 0.81. In addition, we wanted to create a balanced distribution of cases across the sample in order to avoid potential bias from unequal group sizes. Since FTD cases are relatively rare, we chose to limit the sample size to 10 subjects per group.

### Patient selection

Retrospectively, we searched our local PACS for patients who underwent imaging during routine clinical practice between 2011 and 2018, meeting the following inclusion criteria: age over 18, presence of Alzheimer’s dementia (AD) or Frontotemporal dementia (FTD), interdisciplinary clinically confirmed diagnosis in collaboration with psychiatry, interdisciplinary confirmed diagnosis with nuclear medicine (positron emission tomography (PET)-MR), representative regional atrophy pattern in brain MR assessed by a neuroradiology attending, and presence of 1 mm isovoxel 3D T1, 1 mm isovoxel 3D FLAIR and 4 mm coronal T2. For healthy control (HC), the inclusion criteria were: age over 18, absence of dementia or any other severe neurological diagnosis that could alter brain morphology (e.g., stroke, tumor, etc.), unremarkable findings in the report, and presence of 1 mm isovoxel 3D T1, 1 mm isovoxel 3D FLAIR, and 4 mm coronal T2.

### MRI scans and data pre-processing

MRI scans were obtained by healthcare professionals during clinical routine on a 3-T PET-/MRI hybrid scanner (Siemens Biograph). Patients underwent unenhanced imaging, and images comprised of at least 1 mm isovoxel 3D T1w, 1 mm isovoxel 3D FLAIR, secondary reconstructed 4 mm axial FLAIR (from 1 mm isovoxel 3D FLAIR, using PACS), and 4 mm coronal T2. Acquisition parameters are listed in Table 1. After patient selection, images were visually inspected by a neuroradiology resident for image quality. Then, 3D T1, 3D FLAIR, axial FLAIR, and coronal T2 for each patient were exported pseudonymously in the form of Digital Imaging and Communications in Medicine (DICOM) files. DICOM to Neuroimaging Informatics Technology Initiative (NifTI) conversion was performed using dcm2nii [6]. The age of the respective test patient at the time of scanning, as well as the sex, was noted as a relevant covariate for subsequent analyses. If uploading MRI scans to external servers, defacing has become mandatory for anonymization, and was performed using PyDeface [7]—showing negligible impact on atrophy estimation [8].

**Table 1:** Acquisition parameters for the reference standard 3D MPRAGE, as well as the input sequences for the generation of synthetic images with SynthSR acquired at a 3-T scanner (Siemens Biograph)

Acquisition parameter	3D T1	3D FLAIR	Axial FLAIR (secondary reconstruction)	Coronal T2
TR (ms)	2300	5000	5000	9944
TE (ms)	2.98	395	395	109
TI (ms)	900	1800	1800	NA
Slice thickness (mm)	1	1	4	4
Interslice gap (mm)	0	0	0	0.4
Acquisition matrix (freq × phase)	256 × 240	256 × 262	NA (reconstructed from 3D FLAIR)	384 × 384
FOV (mm × mm)	256 × 240	256 × 262	256 × 262	227 × 227
In-plane resolution (mm × mm)	1.0 × 1.0	1.0 × 1.0	0.5 × 0.51*	0.59 × 0.59
Flip angle (°)	9	120	120	165

^*^ Reconstructed resolution based on 512 × 512 matrix over 256 × 262 mm FOV. True resolution is limited by the original 1 mm isotropic 3D FLAIR acquisition

*TR* Repetition time, *TE* Echo time, *TI* Inversion time, *FOV* Field of view, *FLAIR* Fluid-attenuated inversion recovery

### Generation of deep learning-based synthetic images

Deep learning-based synthetic 3D T1 sequences from 1 mm 3D FLAIR, reconstructed 4 mm axial FLAIR, and 4 mm coronal T2 each were generated using the SynthSR tool [5] in FreeSurfer v7.3. [9].

### Nomenclature of synthetic sequences

Even though synthetic imaging is a rapidly developing field, to our knowledge, so far, no standardized nomenclature has been established. To facilitate the understandability and readability of this paper, we propose a nomenclature system that enables straightforward differentiation between acquired and deep learning-based synthetic images.

To address this, we propose the following system:

1. Real vs deep learning-based synthetic Identification: Real images are labeled with an “r,” while deep learning-based synthetic images are labeled with an “s.” For example, a real T1-weighted (T1w) sequence would be denoted as rT1, and a deep learning-based synthetic T1w sequence as sT1.
2. For deep learning-based synthetic sequences, all information about the input sequence should be denoted in superscript.
3. Input sequence specification: a deep learning-based synthetic T1w image derived from a FLAIR sequence would be sT1^FLAIR^, a T1w image derived from a T2 sequence would be sT1^T2^.
4. Orientation details: orientation of the input sequence is indicated using abbreviations: 3D for three-dimensional acquisitions, Ax, Sag, or Cor for axial, sagittal, or coronal orientations, respectively. For instance, a synthetic T1w image generated from a 3D FLAIR input would be labeled as sT1^3DFLAIR^, and one derived from a coronal T2-weighted input would be sT1^CorT2^.
5. Resolution details: resolution is specified using the voxel size, followed by orientation, and the input sequence. E.g., a synthetic T1w image derived from a 4 mm Axial FLAIR would be labeled as sT1^4mmAxFLAIR^, one derived from a 4 mm coronal T2 would be sT1^4mmCorT2^.
6. Optional components like orientation or resolution can be omitted if not relevant in order to keep the terms as short and understandable as possible.

### Automated brain volumetry and disease classification

Automated brain volumetry and disease classification were performed using the AssemblyNet-AD-FTD pipeline in VolBrain [10]. This pipeline employs a deep decision-making process based on two assemblies of 125 3D U-Nets to achieve whole-brain segmentation [11]. It can also detect diseases and differentiate between AD and FTD vs controls by combining structure grading maps of disease-related patterns and structure atrophy, obtained using a support vector machine model (multi-layer perceptron classifier) [12]. To ensure the quality of volumetric segmentations, all segmentations were visually reviewed by a neuroradiology resident. Example images of volumetric segmentations are provided in Suppl. Fig. 1A.

### FreeSurfer volumetry

To support the findings resulting from automated brain volumetry in volBrain, we additionally performed volumetry using *SynthSeg*+ [13] in FreeSurfer v7.4 [9]. This step was included to assess whether the observed volumetric differences for SynthSR-generated sequences were consistent across different segmentation tools, thereby improving generalizability. The standard FreeSurfer recon-all pipeline could not be applied to all synthetic images due to processing failures; therefore, we used SynthSeg, which is robust to variations in contrast and resolution. Analogous to volumetric segmentations resulting from the AssemblyNet-AD-FTD pipeline in VolBrain, volumetric segmentations were subjected to visual quality control by a neuroradiology resident. Example images of volumetric segmentations are provided in Suppl. Fig. 1B.

### Statistical analysis

Data were analyzed, and graphs were generated using R with the packages ggalluvial and ggplot2 [14] and GraphPadPrism (version 10.2.3). To determine the normal distribution of the data, Normality and Lognormality Tests (D’Agostino & Pearson, Anderson–Darling, Shapiro–Wilk, and Kolmogorov–Smirnov tests) were executed, and the predominant outcome was chosen. To compare volumetric measures, as well as disease probabilities between groups, one-way ANOVA—and in cases with missing values, mixed-effects analysis (REML)—was applied, followed by post hoc testing (Dunnett and Tukey).

## Results

### Patient population

**Fig. 1:**
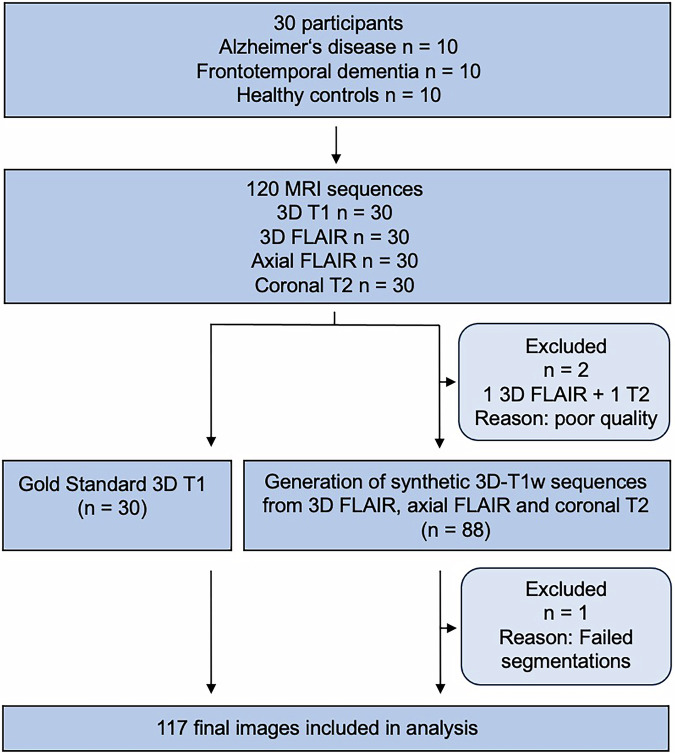
Inclusion and exclusion flowchart. Thirty participants (10 Alzheimer’s disease, 10 FTD, and 10 HC) contributed 120 MRI sequences. After exclusions for poor quality and failed segmentation, 117 images were included in the final analysis

**Fig. 2:**
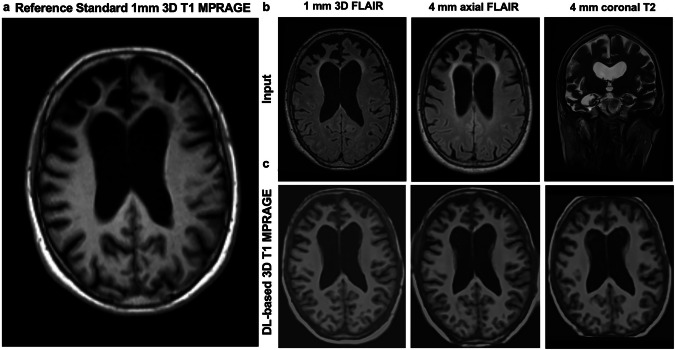
Example of input image quality and partial volume effect. Slices through the brain of a patient with FTD. The reference standard for brain volumetry, real 3D T1 MPRAGE, is shown in **a**. Input sequences (real sequences) used for generating synthetic sequences are displayed in **b** to demonstrate image quality. The corresponding SynthSR-generated 3D T1 sequences are presented in **c**. The partial volume effect of the parenchyma is evident in all SynthSR-generated images, particularly pronounced in the synthesized 3D T1 from coronal T2 input

A total of 30 participants were included in the study (Fig. 1): each 10 patients with AD and FTD, and 10 HCs without clinical or imaging evidence of neurodegenerative disease. From these participants, 120 MRI sequences were initially acquired, including 3D T1w, 3D FLAIR, and coronal T2-weighted images. Following visual inspection, the 3D FLAIR sequence for one AD patient and the coronal T2 sequence for one FTD patient were deemed to be of insufficient quality. Consequently, these specific sequences were excluded from analysis, although the patients themselves remained included in the study. Deep learning-based synthetic 3D T1 sequences from 1 mm 3D FLAIR, reconstructed 4 mm axial FLAIR, and 4 mm coronal T2 were generated and inspected. Example images to demonstrate input image quality are shown in Fig. 2. Synthetic 3D T1w images derived from one T2 sequence of another AD patient showed implausible segmentations across both volumetric tools, leading to exclusion of those segmentations. In total, 117 sequences obtained from thirty participants, including those with AD, FTD, and HC without clinical or imaging evidence of neurodegenerative disease, were included in the study. Mean age was 62.5, 43% were male, 57% were female, for more details see Table 2.

**Table 2:** Patient population of the study

		AD	FTD	HC	Total
Patients	Age range (mean)	46–79 (64.5)	43–72 (60.5)	54–72 (62.5)	43–79 (62.5)
	Female	40%	70%	60%	57%
	Male	60%	30%	40%	43%
	*n* patients	10	10	10	30
Sequences	1 mm isovoxel 3D T1	10	10	10	30
	1 mm isovoxel 3D FLAIR	9	10	10	29
	4 mm axial FLAIR	10	10	10	30
	4 mm coronal T2	9	9	10	28
	*n* sequences	38	39	40	117

*AD* Alzheimer’s dementia, *FTD* Frontotemporal dementia, *HC* Healthy control

### Brain volumetry

#### Global brain volumes

**Fig. 3:**
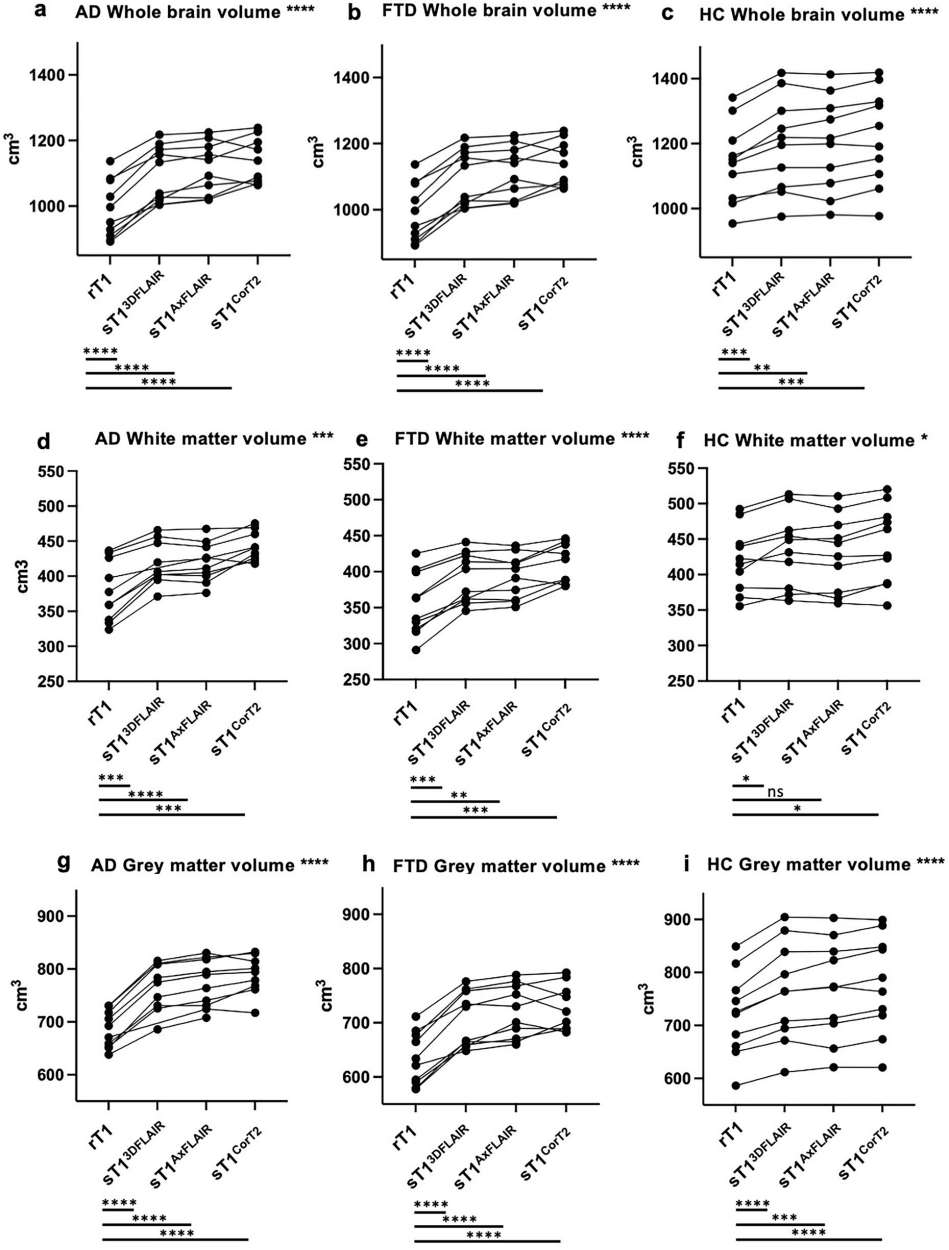
Global brain volumes. Whole brain volume (**a**–**c**), total WM volume (**d**–**f**), and total gray matter volume (**g**–**i**) in the Alzheimer’s Disease (AD) subgroup, in the subgroup of patients with FTD, and in the subgroup of HC. Each dot represents one patient in the respective subgroup resulting from analysis of the real or SynthSR-generated T1s. Lines connect dots from the same patient. Ns, not significant, ^*^p ≤ 0.05, ^**^p ≤ 0.01, ^***^p ≤ 0.001, ^****^p ≤ 0.0001

Whole brain volume (white matter (WM) volume + gray matter (GM) volume, *p* for AD, FTD and HC subgroups < 0.0001), total WM volume (AD *p* = 0.0002, FTD *p* < 0.0001, HC *p* = 0.0116) and total GM volume (*p* for AD, FTD and HC subgroups < 0.0001) differed significantly between real 3D T1 and deep learning-based synthetic sequences in almost all subgroups (Fig. 3a-1 and Suppl. Fig. 2). The only exception was total WM volume in the subgroup of HC, where there was no significant difference between real 3D T1 and deep learning-based synthetic 3D T1 from axial FLAIR input. Mean total volumes and standard deviation, as well as significant difference compared to the reference standard per subgroup and underlying sequence, are shown in Table 3.

**Table 3:** Global brain volumes for reference standard (real T1) and synthetic T1 sequences from the different input sequences in cm^3^ (mean ± SEM)

Volume (cm^3^)	Sequence		Alzheimer’s disease	FTD	HC
Whole brain (WM + GM)	Reference standard		1063 ± 22.96	991 ± 27.86	1141 ± 38.68
	Input sequence for synthetic T1	3D FLAIR	1184 ± 24.29^****^	1096 ± 26.91^****^	1198 ± 46.00^***^
		Axial FLAIR	1192 ± 21.70^****^	1019 ± 25.02^****^	1198 ± 45.89^**^
		Coronal T2	1232 ± 18.32^****^	1141 ± 23.11^****^	1221 ± 46.65^***^
WM	Reference standard		378.5 ± 13.54	361.9 ± 13.25	420.6 ± 14.51
	Input sequence for synthetic T1	3D FLAIR	418.6 ± 10.56^***^	390.6 ± 10.96^***^	435.0 ± 16.67^*^
		Axial FLAIR	419.5 ± 8.90^****^	393.1 ± 9.71^**^	430.7 ± 16.68^ns^
		Coronal T2	442.8 ± 6.97^***^	411.9 ± 9.14^***^	442.8 ± 17.46^*^
GM	Reference standard		684.9 ± 11.00	633.6 ± 15.33	720.7 ± 25.04
	Input sequence for synthetic T1	3D FLAIR	764.7 ± 15.04^****^	705.7 ± 16.14^****^	763.5 ± 29.68^****^
		Axial FLAIR	772.3 ± 14.16^****^	720.2 ± 15.51^****^	767.7 ± 29.44^***^
		Coronal T2	788.7 ± 12.25^****^	729.4 ± 14.13^****^	777.8 ± 29.39^****^

For the synthetic sequences from different inputs, it is also indicated whether the volumes differ significantly from the reference standard

^ns^ not significant

^*^p ≤ 0.05, ^**^p ≤ 0.01, ^***^p ≤ 0.001, ^****^p ≤ 0.0001

**Fig. 4:**
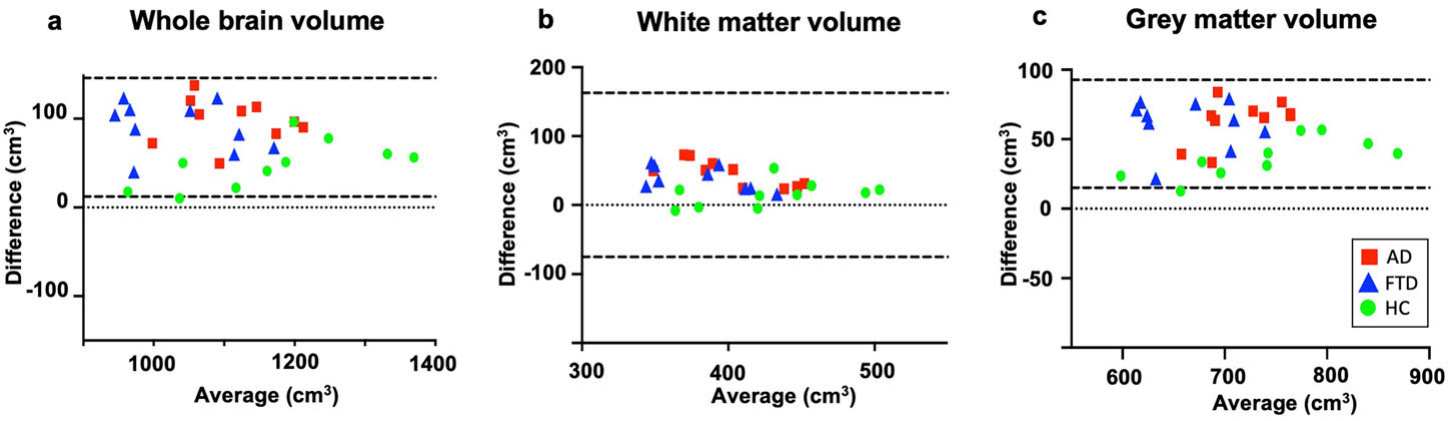
Bland–Altman plots showing the difference in volume estimates (SynthSR-generated 3D T1s from 3D Flair, axial FLAIR, and coronal T2 input vs reference standard 3D T1) plotted against their average (the mean of the two volume measurements). Comparisons are shown for (**a**) whole brain volume, (**b**) WM volume, and (**c**) GM volume. Dashed lines indicate 95% limits of agreement. Color coding indicates diagnostic subgroups: red = Alzheimer’s disease (AD), blue = Frontotemporal dementia (FTD), and green = healthy controls (HC)

Bland–Altman plots (Fig. 4) comparing mean volumes resulting from deep learning-based synthetic 3D T1s from the three different inputs vs the reference standard 3D T1 reveal systematic differences across diagnostic groups. HC shows smaller volume differences than AD and FTD, and spans higher average volume values (defined as the mean of the two measurements). In contrast, AD and FTD subgroups exhibit larger differences and lower average volumes. Overall, differences tend to be positive (> 0), indicating a systematic offset suggesting a consistent overestimation of volumes in SynthSR-generated images.

When examining the individual volumes, 100% of whole brain and GM volumes, as well as 92% of WM volumes resulting from volumetry of deep learning-based synthetic sequences, regardless of the input sequence, were higher than the reference standard. Percentage differences were significantly higher for all global input sequences (Suppl. Table 1).

Although VolBrain has been compared with other volumetric tools demonstrated good reproducibility [10], we also evaluated global volumes using SynthSeg+ in FreeSurfer in order to assess whether the observed volumetric differences for SynthSR-generated sequences were consistent across different segmentation tools, improving generalizability. Volumetric analysis using SynthSeg+ in FreeSurfer yielded similar observations as seen using volBrain: all global volumes for all subgroups resulting from the deep learning-based synthetic 3D T1s were larger compared to the reference standard real 3D T1, with the most pronounced differences observed for 4 mm coronal T2 input, followed by 4 mm axial FLAIR input, and the least pronounced differences for 1 mm 3D FLAIR input (Suppl. Fig. 3 and Suppl. Table 2). Direct volumetric analysis of the input sequences tended to overestimate WM and underestimate GM relative to the reference standard; however, except for one case, whole-brain volume did not differ significantly (Suppl. Table 2). Comparisons between SynthSeg+ volumetry of deep learning-based synthetic 3D T1 images and direct volumetry of their respective input sequences revealed significant differences in whole-brain, GM, and hippocampal volumes for all subgroups, but not in WM volume, for AD and FTD across all sequences and for axial FLAIR in HC (Suppl. Table 3).

#### Regional brain volumes

Matching the results from the global volumes, also for all cerebral lobar volumes, values did significantly differ for all subgroups and all input sequences (frontal lobe for AD, FTD and HC subgroups *p* < 0.0001; temporal lobe AD and HC *p* < 0.0001, FTD *p* = 0.0004; parietal lobe AD *p* < 0.0001, FTD *p* = 0.0002, HC *p* = 0.0001; occipital lobe AD *p* = 0.0003, FTD *p* = 0.0009, HC *p* < 0.0001, Table 4 and Suppl. Fig. 3). Hippocampal total volumes did not differ in the AD, as well as the FTD subgroup, between the reference standard 3D T1 and any of the deep learning-based synthetic 3D T1w images. In the subgroup of HC (*p* < 0.0001) there were significant differences comparing real 3D T1 vs synthetic 3D T1 generated from 3D and axial FLAIR input (sT1^3DFLAIR^ adj. *p* = 0.0252, sT1^AxFLAIR^ adj. *p* = 0.0011), but no significant differences compared to deep learning-based synthetic 3D T1 synthesized from coronal T2 input (Table 4).

**Table 4:** Regional brain volumes for reference standard (real T1) and synthetic T1 sequences from the different input sequences in cm^3^ (mean ± SEM)

Volume (cm^3^)	Sequence		Alzheimer’s disease	FTD	HC
Frontal lobe	Reference standard		178.8 ± 2.65	152.2 ± 7.40	182.9 ± 6.19
	Input sequence for synthetic T1	3D FLAIR	201.4 ± 3.4^****^	178.9 ± 6.77^****^	198.4 ± 7.76^****^
		Axial FLAIR	205.9 ± 3.35^****^	185.8 ± 6.80^****^	201.9 ± 7.79^****^
		Coronal T2	207.5 ± 2.57^****^	189.6 ± 5.24^****^	200.3 ± 8.08^***^
Temporal lobe	Reference standard		99.31 ± 2.65	97.69 ± 4.18	114.2 ± 4.41
	Input sequence for synthetic T1	3D FLAIR	117.7 ± 2.91^****^	114.4 ± 3.17^**^	122.9 ± 5.20^****^
		Axial FLAIR	121.4 ± 3.13^****^	117.9 ± 3.01^***^	124.9 ± 5.13^****^
		Coronal T2	121.7 ± 2.97^****^	117.7 ± 3.68^****^	126.7 ± 5.11^****^
Parietal lobe	Reference standard		100.5 ± 2.88	101.6 ± 2.78	113.4 ± 3.91
	Input sequence for synthetic T1	3D FLAIR	112.4 ± 3.15^****^	109.1 ± 2.74^***^	119.5 ± 4.77^***^
		Axial FLAIR	115.6 ± 2.95^****^	111.8 ± 2.24^***^	120.6 ± 4.72^**^
		Coronal T2	120.0 ± 2.84^****^	112.7 ± 2.81^****^	120.6 ± 4.76^**^
Occipital lobe	Reference standard		87.65 ± 2.07	83.77 ± 2.17	86.00 ± 3.80
	Input sequence for synthetic T1	3D FLAIR	97.51 ± 2.04^****^	89.32 ± 2.06^**^	90.17 ± 4.27^**^
		Axial FLAIR	94.98 ± 2.25^***^	89.30 ± 2.01^**^	90.02 ± 4.17^**^
		Coronal T2	96.02 ± 2.47^**^	89.18 ± 2.44^**^	91.96 ± 3.92^***^
Hippocampus	Reference standard		6.49 ± 0.20	5.60 ± 0.33	7.17 ± 0.27
	Input sequence for synthetic T1	3D FLAIR	6.42 ± 0.18^ns^	5.77 ± 0.16^ns^	6.82 ± 0.24^*^
		Axial FLAIR	6.22 ± 0.17^ns^	5.81 ± 0.22^ns^	6.40 ± 0.21^**^
		Coronal T2	6.94 ± 0.22^ns^	6.24 ± 0.15^ns^	6.92 ± 0.27^ns^

For the synthetic sequences from different inputs, it is also indicated whether the volumes differ significantly from the reference standard

^Ns^ not significant

^*^p ≤ 0.05, ^**^p ≤ 0.01, ^***^p ≤ 0.001, ^****^p ≤ 0.0001

In the Bland Altman plot, differences, as well as the mean of differences of lobar brain volumes (Suppl. Fig. 4) tend to be positive, similarly to the global volumes, indicating a systematic offset that suggests an overestimation of volumes in the deep learning-based synthetic 3D T1w sequences. Only for hippocampus total volume, means are distributed about equally around zero (Suppl. Fig. 4), not indicating a systemic offset.

Examination of the individual volumes revealed 100% of frontal and temporal volumes, as well as > 90% of parietal and occipital lobe volumes, resulting from volumetry of deep learning-based synthetic sequences being higher than the reference standard, regardless of the input sequence. Percentage differences were—except for Hippocampus—significantly higher for all regional volumes for all input sequences. For Hippocampus, in total 41% of volumes from deep learning-based synthetic sequences were larger than the reference standard, with no significant percentage deviations for deep learning-based synthetic 3D T1 synthetized from 3D FLAIR and axial FLAIR input (Supplementary Table 1).

Hippocampus volumetry from analysis with SynthSeg+ resulted in higher volumes for some deep learning-based synthetic images (sT1^CorT2^ in the AD subgroup, sT1^3DFLAIR^ and sT1^CorT2^ in the FTD and HC subgroup, Suppl. Fig. 2j-l and Suppl. Table 2). Lobar volumes were not evaluated for SynthSeg+ due to their absence as direct outputs and to ensure comparability.

### Automated disease probability prediction

**Fig. 5:**
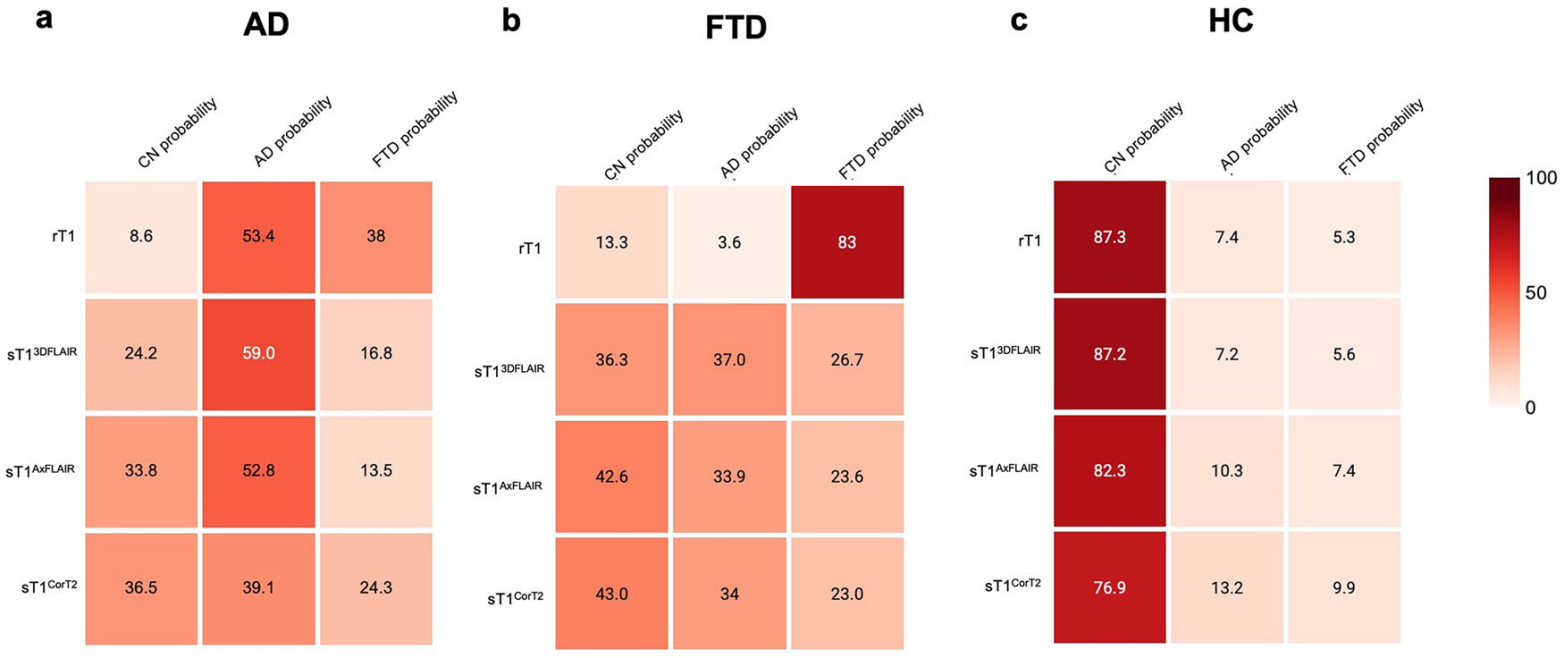
Automatic disease probability prediction. Alzheimer’s Disease (AD) probability in the subgroup of patients diagnosed with AD (**a**), FTD probability in the subgroup of patients diagnosed with FTD (**b**), and Cognitively Normal (CN) probability in the subgroup of HC (**c**). Each dot represents one patient in the respective subgroup resulting from analysis of the real or SynthSR-generated T1s. Lines connect dots from the same patient. Values result from automated brain atrophy estimation from real 3D T1 (reference standard), as well as SynthSR-generated 3D T1 from different inputs (3D FLAIR, axial FLAIR, coronal T2). ns = not significant, ^*^p ≤ 0.05, ^**^p ≤ 0.01, ^***^p ≤ 0.001, ^****^p ≤ 0.0001. Results from automated disease probability prediction using the AssemblyNet-AD-FTD in volBrain, depending on the input sequence for each case, result in a total percentage of 100%, which is divided into percentages for CN, AD, and FTD for AD subgroup (**a**), FTD subgroup (**b**), and HC (**c**)

To identify possible effects on automated disease classification of a real or deep learning-based synthetic image as Alzheimer’s Disease, FTD, or cognitively normal, we compared automated disease probabilities from real and SynthSR-generated sequences resulting from analysis with the AssemblyNet-AD-FTD pipeline from VolBrain [3]. The AssemblyNet-AD-FTD pipeline computes disease probability prediction automatically in a deep decision-making process by combining structure grading maps of disease-related patterns and structure atrophy by using a support vector machine model (multi-layer perceptron classifier). It provides a percentage probability for AD, FTD, and Cognitively Normal status, which together always total 100%. The resulting mean percentages per subgroup and input sequence are shown in Fig. 5.

Comparing automated disease probabilities from the subgroup of patients diagnosed with Alzheimer’s Disease, no notable variances in AD probability between the reference standard (rT1) and deep learning-based synthetic 3D-T1 generated from different inputs were revealed. Comparison of automated disease probabilities from the subgroup of patients diagnosed with FTD revealed significant differences in FTD probability between deep learning-based synthetic 3D-T1s from all inputs, with pronouncedly lower values for the deep learning-based synthetic sequences. Categorization as cognitively normal in the subgroup of HC showed significant differences depending on the input sequence (*p*-Value = 0.0007). Whereas no significant differences were observed for sT1^3DFLAIR^ and sT1^AxFLAIR^, a significant difference was observed for sT1^CorT2^ (adj. *p* = 0.0054), see also Table 5.

**Table 5:** Automated disease probabilities in the corresponding subgroup

Disease probabilities in %		AD	FTD	Cognitively normal
Reference standard		53.41 ± 8.89%	83.1 ± 9.68%	87.28 ± 4.15%
Input sequence for synthetic T1	3D FLAIR	58.97 ± 5.84%^ns^	26.72 ± 7.21%^***^	87.2 ± 3.13%^ns^
	Axial FLAIR	52.75 ± 6.05%^ns^	23.58 ± 5.27%^***^	82.26 ± 3.34%^ns^
	Coronal T2	42.74 ± 4.75%^ns^	22.99 ± 3.39%^***^	76.89 ± 3.59%^**^

I.e., Alzheimer’s disease probability in the subgroup of patients with AD, FTD probability for FTD in the subgroup of patients with FTD, and cognitively normal probability in the subgroup of HC

Stars indicate whether the volumes differ significantly from the reference standard

^Ns^ not significant

^*^p ≤ 0.05, ^**^p ≤ 0.01, ^***^p ≤ 0.001, ^****^p ≤ 0.0001

For the reference standard T1, automated disease classification generally performs well. However, for deep learning-based synthetic T1s, regardless of the input sequences, the classification as FTD significantly declines. In these instances, cases are typically reclassified with the highest percentage as either AD or healthy when deep learning-based synthetic T1s are investigated. While classification as healthy is effective, it tends to be overly sensitive, often misclassifying FTD cases with very high percentages as cognitively normal in the synthetic sequences. This tendency becomes more pronounced when synthetic T1 is generated from coronal T2 input.

## Discussion

In this study, we systematically evaluated volumetric measurements based on deep learning-based synthetic 3D T1w sequences generated from various inputs in HC and patients with region-specific atrophy patterns. We found systematic errors in global and regional brain volumetric measurements, resulting in overestimated volumes compared to the reference standard 3D T1. Interestingly, we did not find especially pronounced effects for regions with disease-specific atrophy, e.g., effects were not more prominent in frontal or temporal lobes in FTD than in parietal or occipital lobes. Last, we show preliminary evidence that using deep learning-based synthetic 3DT1 imaging as input alters a deep learning algorithm for automated disease classification.

**Fig. 6:**
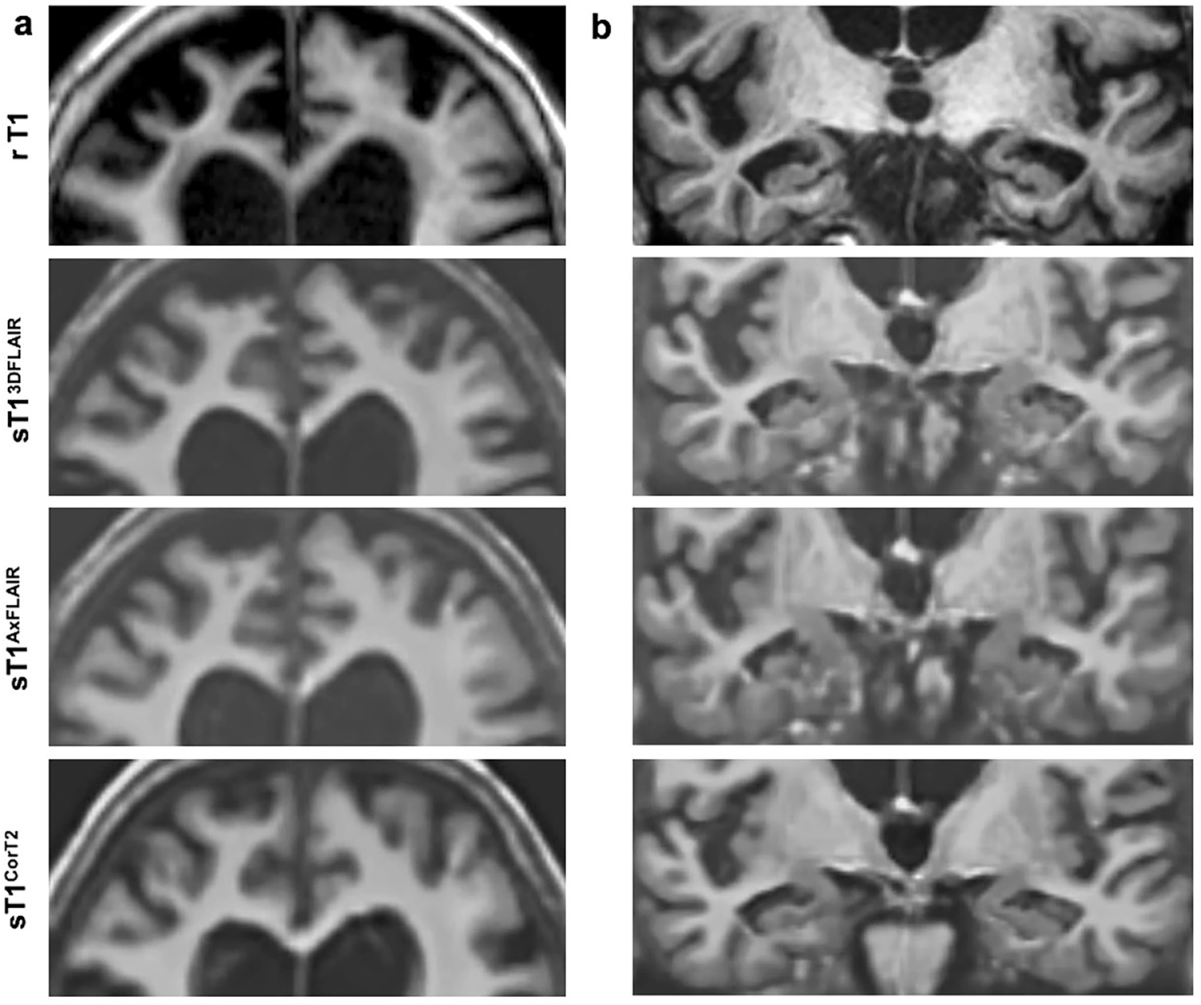
Real 3D T1 and SynthSR generated 3D T1w images from the different input sequences of the frontal lobe of one patient with FTD (**a**) and the Hippocampus from one patient with Alzheimer’s Disease (**b**). In (**a**) partial volume effect of the parenchyma is evident in all SynthSR-generated images, particularly pronounced in  sT1^corT2^. rT1, real 3D T1; sT1^3DFLAIR^, SynthSR generated 3D T1 from 3D FLAIR input; sT1^AxFLAIR^, SynthSR generated 3D T1 from axial FLAIR input; sT1^CorT2^, SynthSR generated 3D T1 from coronal T2 input

Almost all global and regional volumes resulting from the different deep learning-based synthetic sequences were larger than the corresponding values resulting from the reference standard 3D T1, being slightly more pronounced for the coronal T2. In a rough approximation, deep learning-based synthetic volumes were about 10% larger than the reference standard, with the biggest differences for the frontal and temporal lobes and the smallest for the occipital lobe. One reason for this overestimation may stem from partial volume effects, mainly pronounced in the coronal T2 (Fig. 6a). This effect arises when a single voxel contains signals from multiple tissues, leading to blurred tissue boundaries in the image. It is a well-known phenomenon that impacts morphometry, and while various correction methods have been proposed, a slight residual effect is likely unavoidable [15, 16]. Incorporating input-specific characteristics into the generation process could help mitigate such issues.

Domain shift—variations in image characteristics or data distribution that arise when imaging is conducted across different scanners, protocols, or populations—likely contributes to inconsistencies. These can be diminished by incorporating deep learning-based synthetic images for training or employing domain adaptation (DA) methods to address domain shift [17–19]. While SynthSeg+ utilizes a domain randomization strategy and was trained on randomized, unrealistic synthetic data, the tendency for volumes to be overestimated persists.

SynthSR was trained on datasets with various diseases containing brain tumors, strokes, and Alzheimer’s disease, but in the context of neurodegenerative diseases primarily validated on hippocampal volumes in an AD cohort. This may explain its better performance on hippocampal volumes (Fig. 6b), as well as in AD classification compared to rarer dementias like FTD. On the other hand, validation via intraclass correlation (ICC), resulting in systematic errors of absolute values [5], could potentially explain the systematic shift in representation of brain volumes.

Input quality—contrast, resolution, and orientation—clearly seems to influence volumetric accuracy. Overestimation was highest with low-resolution coronal T2 and lowest with isovoxel 3D FLAIR. This pattern is observed for both global brain volumes and regional brain volumes, excluding hippocampal volume. Disease-specific atrophy patterns, such as those in FTD, did not appear to amplify errors in affected regions, suggesting no evidence that the volumes obtained from deep learning-based synthetic T1s shifted predominantly towards the “norm.”

Systematic volumetric errors were sufficient to affect automated disease classification, particularly in FTD. In addition to volume discrepancies, subtle differences in image characteristics—possibly even specific to deep-learning synthetic sequences—may also contribute to misclassification. To improve reliability and reduce bias, it is essential to include synthetic data during algorithm training. While deep learning-based synthetic imaging is increasingly used to model disease progression and support diagnostics, the evaluation of prognostic capabilities for deep learning-based synthetic sequences are limited. Automated classification tools show promising accuracy [10, 20–24], and systems like AssemblyNet-AD-FTD offer intuitive, radiology-style reports.

### Study limitations

The relatively small sample size may restrict the statistical power of the findings, limiting the ability to detect potentially significant, more subtle differences or falsely significant results influenced by other factors, and reducing the generalizability of the results. The retrospective nature of the study may introduce selection bias. Apart from this, the patient population, which comprises different age groups and both sexes, shows a lack of diversity, which may introduce bias and limit the applicability of the findings to broader, more diverse populations. This homogeneity can skew results and fails to account for other influencing factors, like genetic factors, environmental or and socio-economic variations present in other racial and ethnic groups. Additionally, the 4 mm axial FLAIR sequence was secondarily reconstructed from a high-resolution 1 mm 3D T1w image. While this approach ensured consistent acquisition parameters and minimized variability, it may not fully capture the quality degradation typically seen in clinically acquired low-resolution FLAIR images and limit the generalizability of our findings. Furthermore, due to investigation and use of recent work on emerging fields due to the rapid evolvement of deep learning-based synthetic imaging, as well as automated brain volumetry and automated disease classification, there is paucity of robust evaluation of the tools, as well as only few previous research on the topic which presents a challenge, as it limits the context and foundation upon which to verify, build and compare our findings. Furthermore, future studies should explore more direct approaches to evaluating sequences other than 3D T1 for volumetric analysis.

## Conclusion

Our data shows that volumetric measurements derived from deep learning-based synthetic images are prone to systematic errors, leading to overestimated global and regional volumes, both in patients with disease-specific atrophy patterns and in HC. Thus, introducing deep-learning-based synthetic imaging sequences into automated volumetric analyses warrants caution. Continued validation in large, diverse cohorts is essential to fully realize its potential for both research and clinical practice.

## Supplementary information

ELECTRONIC SUPPLEMENTARY MATERIAL

## Data Availability

Datasets and analyses are available from the corresponding author upon reasonable request.

## Abbreviations

3D: Three-dimensional
AD: Alzheimer’s dementia
AI: Artificial intelligence
ANOVA: Analysis of variance
DICOM: Digital imaging and communications in medicine
FLAIR: Fluid-attenuated inversion recovery
FTD: Frontotemporal dementia
GM: Gray matter
HC: Healthy control
MPRAGE: Magnetization prepared—rapid gradient echo
MRI: Magnetic resonance imaging
ns: Not significant
PET: Positron emission tomography
SEM: Standard error of the mean
T1w: T1-weighted
WM: White matter

## References

[1] Maresova P, Hruska J, Klimova B, Barakovic S, Krejcar O (2020) Activities of daily living and associated costs in the most widespread neurodegenerative diseases: a systematic review. Clin Interv Aging 15:1841–1862 10.2147/CIA.S264688 PMC7538005 33061334

[2] Nichols E, Steinmetz JD, Vollset SE et al (2022) Estimation of the global prevalence of dementia in 2019 and forecasted prevalence in 2050: an analysis for the Global Burden of Disease Study 2019. Lancet Public Health 7:e105–e125 34998485 10.1016/S2468-2667(21)00249-8 PMC8810394

[3] Graf R, Platzek PS, Riedel EO et al (2025) Generating synthetic high-resolution spinal STIR and T1w images from T2w FSE and low-resolution axial Dixon. Eur Radiol 35:1761–1771 39231829 10.1007/s00330-024-11047-1 PMC11913981

[4] Schlaeger S, Drummer K, El Husseini M et al (2023) Synthetic T2-weighted fat sat based on a generative adversarial network shows potential for scan time reduction in spine imaging in a multicenter test dataset. Eur Radiol 33:5882–5893 36928566 10.1007/s00330-023-09512-4 PMC10326102

[5] Iglesias JE, Billot B, Balbastre Y et al (2023) SynthSR: a public AI tool to turn heterogeneous clinical brain scans into high-resolution T1-weighted images for 3D morphometry. Sci Adv 9:eadd3607 36724222 10.1126/sciadv.add3607 PMC9891693

[6] Li X, Morgan PS, Ashburner J, Smith J, Rorden C (2016) The first step for neuroimaging data analysis: DICOM to NIfTI conversion. J Neurosci Methods 264:47–56 26945974 10.1016/j.jneumeth.2016.03.001

[7] Gulban OF, Nielson D, J Lee (2022) poldracklab/pydeface: PyDeface v2.0.2. *Zenodo*10.5281/zenodo.6856482

[8] Rubbert C, Wolf L, Turowski B et al (2022) Impact of defacing on automated brain atrophy estimation. Insights Imaging 13:54 35348936 10.1186/s13244-022-01195-7 PMC8964867

[9] Fischl B (2012) FreeSurfer. Neuroimage 62:774–781 22248573 10.1016/j.neuroimage.2012.01.021 PMC3685476

[10] Manjón JV, Coupé P (2016) volBrain: an online MRI brain volumetry system. Front Neuroinform 10:30 27512372 10.3389/fninf.2016.00030 PMC4961698

[11] Coupé P, Mansencal B, Clément M et al (2020) AssemblyNet: a large ensemble of CNNs for 3D whole brain MRI segmentation. Neuroimage 219:117026 32522665 10.1016/j.neuroimage.2020.117026

[12] Nguyen HD, Clément M, Planche V, Mansencal B, Coupé P (2023) Deep grading for MRI-based differential diagnosis of Alzheimer’s disease and frontotemporal dementia. Artif Intell Med 144:102636 37783553 10.1016/j.artmed.2023.102636 PMC10904714

[13] Billot B, Magdamo C, Cheng Y, Arnold SE, Das S, Iglesias JE (2023) Robust machine learning segmentation for large-scale analysis of heterogeneous clinical brain MRI datasets. Proc Natl Acad Sci USA 120:e2216399120 36802420 10.1073/pnas.2216399120 PMC9992854

[14] Team RC (2022) R: a language and environment for statistical computing. R Foundation for Statistical Computing, Vienna

[15] Tohka J (2014) Partial volume effect modeling for segmentation and tissue classification of brain magnetic resonance images: a review. World J Radiol 6:855–864 25431640 10.4329/wjr.v6.i11.855 PMC4241492

[16] Meltzer CC, Kinahan PE, Greer PJ et al (1999) Comparative evaluation of MR-based partial-volume correction schemes for PET. J Nucl Med 40:2053–2065 10616886

[17] Kondrateva E, Pominova M, Popova E, Sharaev M, Bernstein A, Burnaev E (2021) Domain shift in computer vision models for MRI data analysis: an overview. SPIE

[18] Zalevskyi V, Sanchez T, Roulet M et al (2024) Improving cross-domain brain tissue segmentation in fetal MRI WITH SYNTHETIC DAta. Springer Nature Switzerland, Cham, pp 437–447

[19] Full PM, Isensee F, Jäger PF, Maier-Hein K (2021) Studying robustness of semantic segmentation under domain shift in cardiac MRI. Springer International Publishing, Cham, pp 238–249

[20] Basaia S, Agosta F, Wagner L et al (2019) Automated classification of Alzheimer’s disease and mild cognitive impairment using a single MRI and deep neural networks. Neuroimage Clin 21:101645 30584016 10.1016/j.nicl.2018.101645 PMC6413333

[21] Klöppel S, Stonnington CM, Chu C et al (2008) Automatic classification of MR scans in Alzheimer’s disease. Brain 131:681–689 18202106 10.1093/brain/awm319 PMC2579744

[22] Zarei A, Keshavarz A, Jafari E et al (2024) Automated classification of Alzheimer’s disease, mild cognitive impairment, and cognitively normal patients using 3D convolutional neural network and radiomic features from T1-weighted brain MRI: a comparative study on detection accuracy. Clin Imaging 115:110301 39303405 10.1016/j.clinimag.2024.110301

[23] El-Assy AM, Amer HM, Ibrahim HM, Mohamed MA (2024) A novel CNN architecture for accurate early detection and classification of Alzheimer’s disease using MRI data. Sci Rep 14:3463 38342924 10.1038/s41598-024-53733-6 PMC10859371

[24] Qiu S, Joshi PS, Miller MI et al (2020) Development and validation of an interpretable deep learning framework for Alzheimer’s disease classification. Brain 143:1920–1933 32357201 10.1093/brain/awaa137 PMC7296847

