# Supplementary material for: Deep learning-based synthetic brain MRI for the assessment of regional atrophy patterns in neurodegenerative diseases
Source: Eur Radiol. 2026 Feb 27;36(7):6025–37. doi: 10.1007/s00330-025-12302-9 (PMC13282297; doi:10.1007/s00330-025-12302-9)
Supplement: Supplementary file 1 — ELECTRONIC SUPPLEMENTARY MATERIAL [file 330_2025_12302_MOESM1_ESM.pdf]

Deep learning-based synthetic brain MRI for the assessment of regional atrophy patterns in neurodegenerative diseases.

ELECTRONIC SUPPLEMENTARY MATERIAL

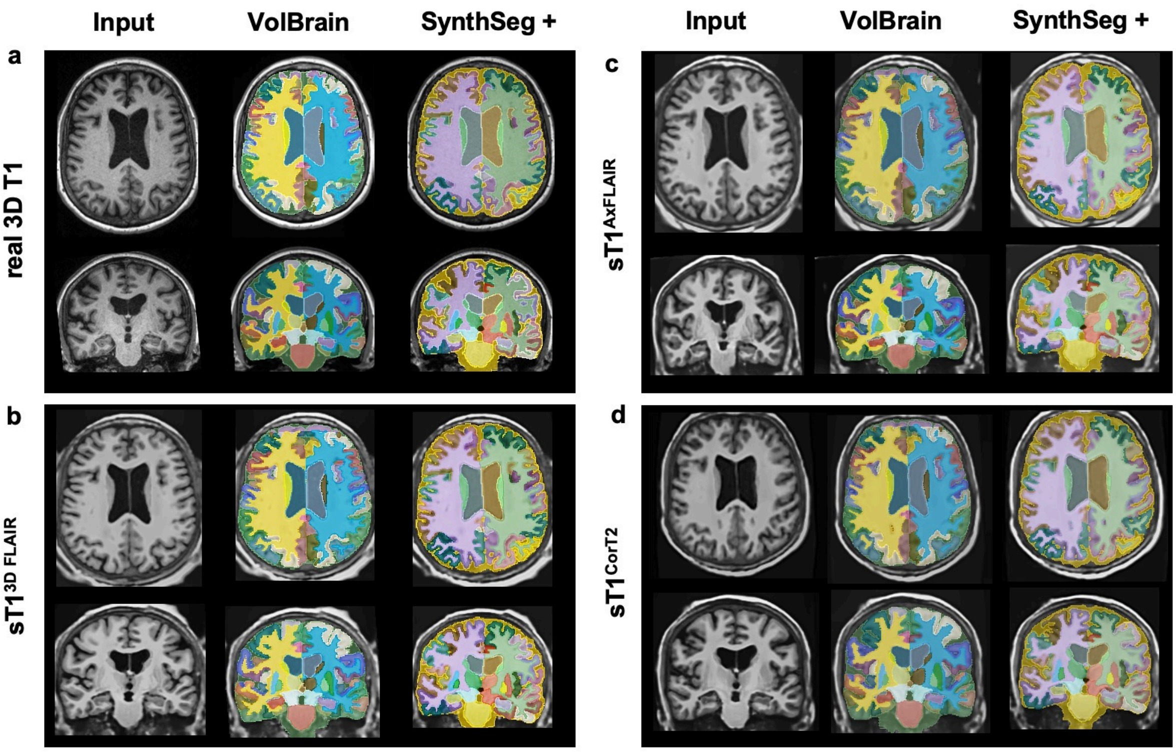

Supplementary Figure 1: Representative example of automated volumetric brain segmentations using the AssemblyNet-AD-FTD pipeline in VolBrain and SynthSeg+ in FreeSurfer. Axial slices from a healthy control subject are shown. The reference standard 3D T1-weighted MPRAGE image **(a)**, along with corresponding deep learning–generated synthetic 3D T1-weighted images from various input modalities **(b-d)**, are presented with their respective volumetric segmentations overlaid.

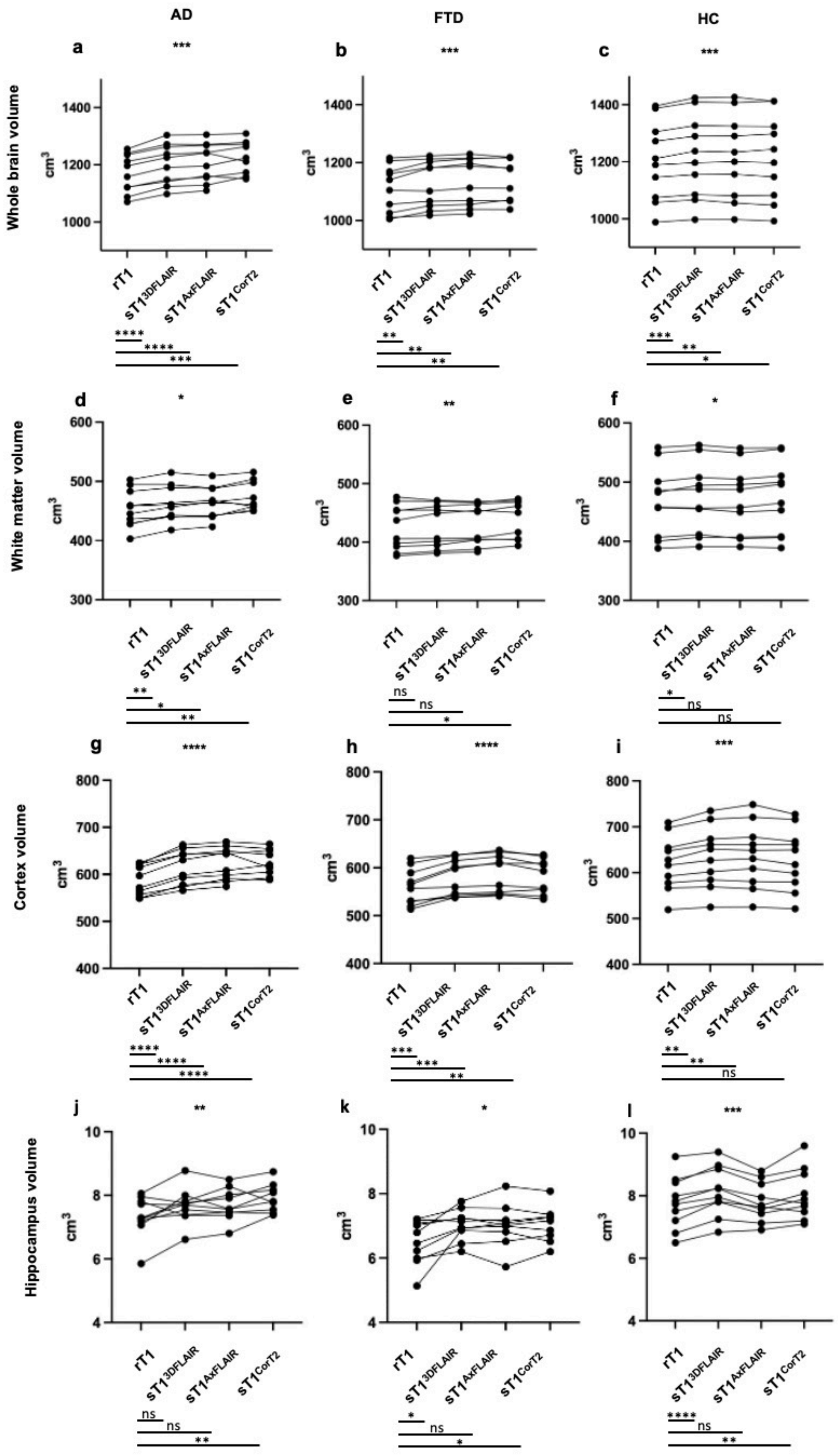

Supplementary Figure 2: Global brain volumes and Hippocampus volumes obtained with SynthSeg+. Whole brain volume (**a-c**), total white matter volume (**d-f**), total cortex volume (**g-i**) and total Hippocampus volume (**j-l**) in the Alzheimer's Disease (AD) subgroup, in the subgroup of patients with Frontotemporal Dementia (FTD) and in the subgroup of Healthy Controls (HC). Each dot represents one patient in the respective subgroup resulting from analysis of the real or SynthSR generated T1w images. Lines connect dots from the same patient. Ns = not significant, * = p ≤ 0.05, ** = p ≤ 0.01, *** = p ≤ 0.001, **** = p ≤ 0.0001.

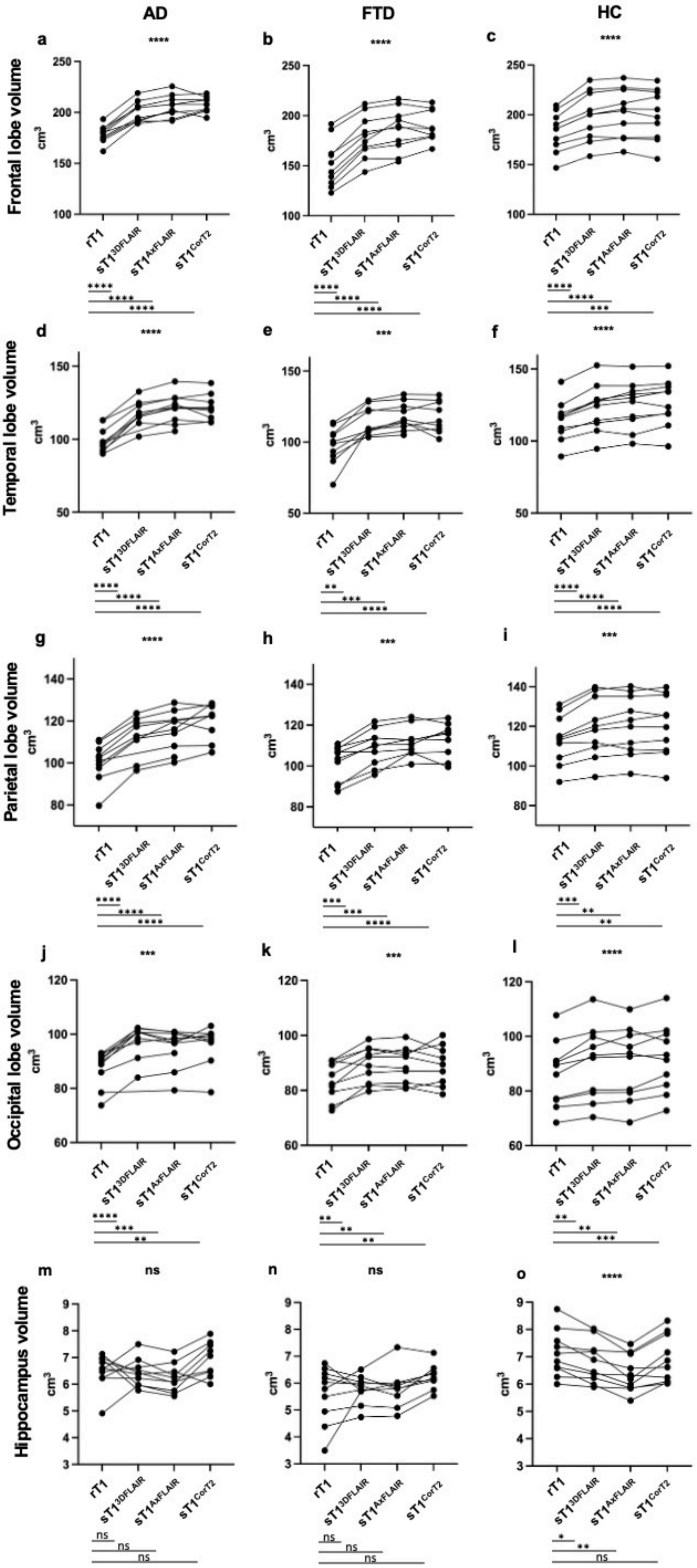
AD
FTD
HC
Frontal lobe volume
Temporal lobe volume
Parietal lobe volume
Occipital lobe volume
Hippocampus volume
cm3
rT1
sT1 3DFLAIR
sT1 AxFLAIR
sT1 CorT2
a
b
c
d
e
f
g
h
i
j
k
l
m
n
o
ns

Supplementary Figure 3: Regional brain volumes. Frontal lobe total volume (**a-c**), temporal lobe total volume (**d-f**), parietal lobe total volume (**g-i**), occipital lobe total volume (**j-l**) and hippocampus total volume (**m-o**) in the subgroup of patients with Alzheimer‘s dementia (AD), in the subgroup of patients with Frontotemporal Dementia (FTD) and in the subgroup of Healthy Controls (HC). Each dot represents one patient in the respective subgroup resulting from analysis of the real or SynthSR generated T1. Lines connect dots from the same patient. Ns = not significant, * = $p \leq 0.05$, ** = $p \leq 0.01$, *** = $p \leq 0.001$, **** = $p \leq 0.0001$.

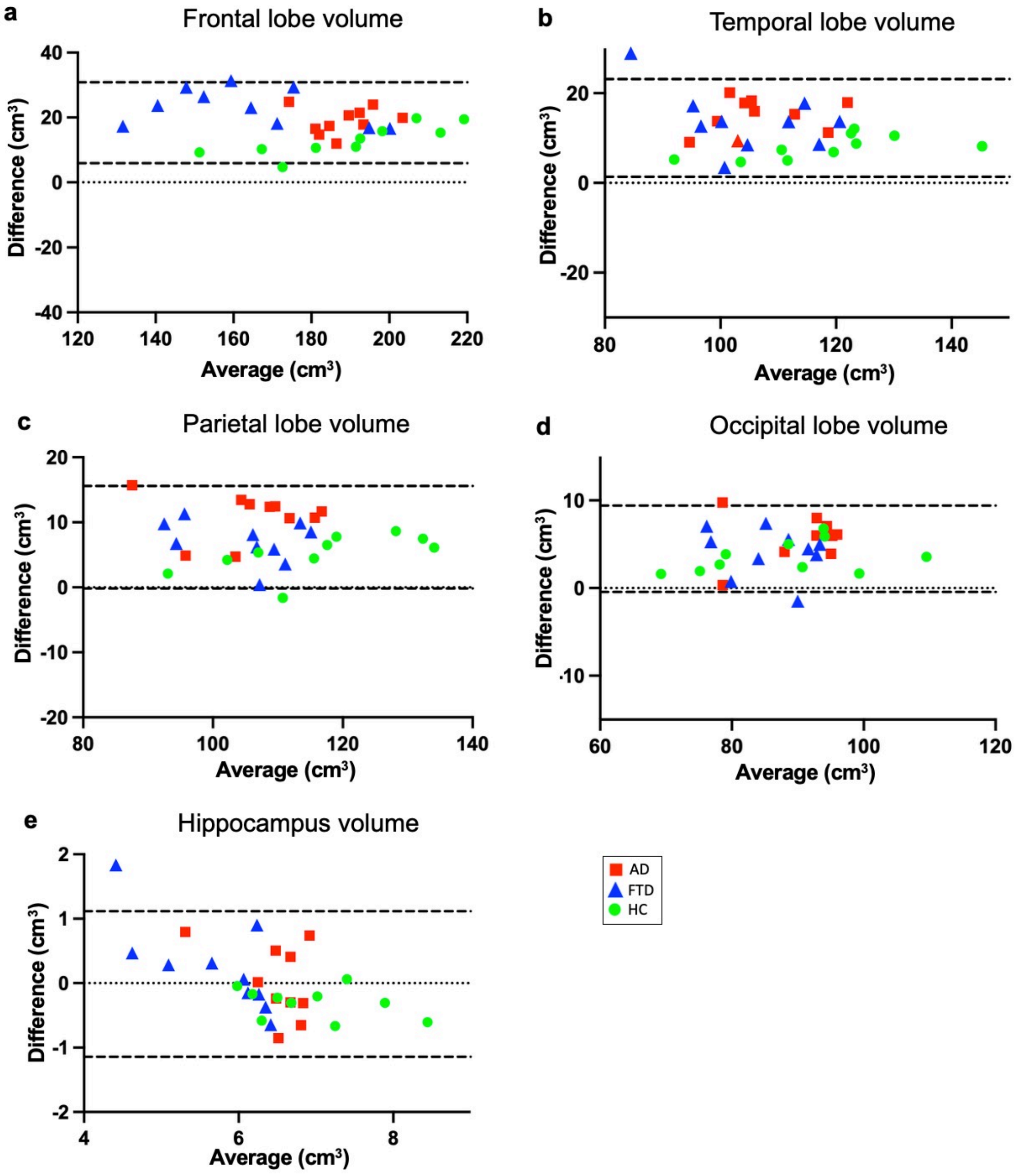

Supplementary Figure 4: Bland-Altman Plots (difference versus average) comparing the mean volumes resulting from the SynthSR generated 3D T1s from the three different inputs (3D Flair, axial FLAIR and coronal T2) versus the reference standard 3D T1 for frontal lobe total volume **(a)**, temporal lobe total volume **(b)**, parietal lobe total volume **(c)** occipital lobe total volume **(d)** and hippocampus total volume **(e).** Dashed lines indicate 95 % confidence intervals. Red = AD subgroup, blue = FTD subgroup, green = healthy controls.

Volume	Sequence		Number of synth. T1s with higher volume than Gold Standard	Difference of volumes of synth. T1s compared to Gold Standard in %. Mean ± SD
Whole brain	Input Sequence for synthetic T1	3D FLAIR	29/29 = 100 %	9.1 ± 2.7 ****
		Axial FLAIR	30/30 = 100 %	9.9 ± 3.7 ****
		Coronal T2	28/28 = 100 %	12.2 ± 4.5 ****
		Total	**87/87 = 100 %**	**10.4 ± 3.6**
White matter	Input Sequence for synthetic T1	3D FLAIR	26/29 = 90 %	8.5 ± 4.7 ****
		Axial FLAIR	27/30 = 90 %	8.4 ± 5.7 ****
		Coronal T2	27/28 = 96 %	12.4 ± 7.7 ****
		Total	**80/87 = 92 %**	**9.8 ± 6.0**
Grey matter	Input Sequence for synthetic T1	3D FLAIR	29/29 = 100 %	9.5 ± 2.5 ****
		Axial FLAIR	30/30 = 100 %	11.0 ± 3.3 ****
		Coronal T2	28/28 = 100 %	12.5 ± 3.4 ****
		Total	**87/87 = 100 %**	**11.0 ± 3.0**
Frontal lobe	Input Sequence for synthetic T1	3D FLAIR	29/29 = 100 %	13.1 ± 3.3 ****
		Axial FLAIR	30/30 = 100 %	16.1 ± 4.3 ****
		Coronal T2	28/28 = 100 %	16.1 ± 5.8 ****
		Total	**87/87 = 100 %**	**15.1 ± 4.4**
Temporal lobe	Input Sequence for synthetic T1	3D FLAIR	29/29 = 100 %	14.8 ± 7.3 ****
		Axial FLAIR	30/30 = 100 %	18.0 ± 8.7 ****
		Coronal T2	28/28 = 100 %	18.0 ± 6.0 ****
		Total	**87/87 = 100 %**	**17.0 ± 7.3**
Parietal lobe	Input Sequence for synthetic T1	3D FLAIR	28/29 = 97 %	8.3 ± 3.5 ****
		Axial FLAIR	29/30 = 97 %	10.6 ± 5.3 ****
		Coronal T2	27/28 = 96 %	12.3 ± 5.1 ****
		Total	**84/87 = 97 %**	**10.4 ± 4.6**
Occipital lobe	Input Sequence for synthetic T1	3D FLAIR	28/29 97 %	7.2 ± 3.5 ****
		Axial FLAIR	29/30 = 97 %	6.6 ± 4.2 ****
		Coronal T2	26/28 = 93 %	8.0 ± 4.7 ****
		Total	**83/87 = 95 %**	**7.3 ± 4.1**
Hippocampus	Input Sequence for synthetic T1	3D FLAIR	10/29 = 34 %	0.9 ± 12.9 ns
		Axial FLAIR	9/30 = 30 %	-2.0 ± 14.4 ns
		Coronal T2	17/28 = 61 %	7.9 ± 14.2 *
		Total	**36/87 = 41 %**	**2.3 ± 13.8**

Supplementary Table 1: Number of synthetic T1s with higher volume than Reference Standard and difference of volumes of synthetic T1s compared to Reference Standard in percent per region and input sequence. Volumes resulting from volumetry of SynthSR generated sequences were compared with the volume from 3D T1 Reference Standard from the same patient. Stars indicate significance from zero resulting from one-sided paired t-test. Ns = not significant, * = p ≤ 0.05, ** = p ≤ 0.01, *** = p ≤ 0.001, **** = p ≤ 0.0001.

Volume (cm^3)	Sequence		Alzheimer's Disease	Frontotemporal Dementia	Healthy Controls
Whole brain	Reference Standard		**1170 ± 21.23**	**1110 ± 25.54**	**1203 ± 43.88**
	Real sequence	3D FLAIR	1166 ± 26.02 ns	1109 ± 27.92 ns	1209 ± 45.42 ns
		Axial FLAIR	1161 ± 22.85 ns	1091 ± 26.56 **	1197 ± 45.91 ns
		Coronal T2	1094 ± 9947 ns	1121 ± 25.03 ns	1199 ± 46.02 ns
	Synthetic T1 generated from input	3D FLAIR	1207 ± 23.70 ****	1127 ± 25.72 *	1219 ± 45.98 ***
		Axial FLAIR	1208 ± 21.13 ****	1134 ± 26.08 **	1217 ± 46.38 **
		Coronal T2	1227 ± 19.19 ***	1145 ± 24.23 *	1216 ± 46.85 *
White matter	Reference Standard		**454.0 ± 10.12**	**424.6 ± 12.10**	**468.6 ± 18.65**
	Real sequence	3D FLAIR	463.6 ± 12.47 **	437.5 ± 13.37 **	485.4 ± 20.22 ***
		Axial FLAIR	458.0 ± 10.02 ns	425.9 ± 12.27 ns	475.5 ± 20.01 *
		Coronal T2	430.3 ± 43.64 ns	441.4 ± 11.64 ***	479.0 ± 19.86 **
	Synthetic T1 generated from input	3D FLAIR	461.9 ± 10.48 **	427.5 ± 11.68 ns	472.8 ± 18.91 *
		Axial FLAIR	463.0 ± 8.44 *	429.6 ± 11.09 ns	470.3 ± 18.61 ns
		Coronal T2	474.2 ± 8.30 **	438.5 ± 10.99 *	474.1 ± 19.15 ns
Cortex	Reference Standard		**587.0 ± 10.08**	**560.4 ± 11.83**	**620.8 ± 18.73**
	Real sequence	3D FLAIR	574.9 ± 12.36 **	547.3 ± 13.98 **	611.5 ± 19.24 **
		Axial FLAIR	579.1 ± 11.52 ns	545.6 ± 13.05 **	611.2 ± 19.99 *
		Coronal T2	548.5 ± 43.18 ns	554.4 ± 12.38 **	606.7 ± 19.74 ***
	Synthetic T1 generated from input	3D FLAIR	618.7 ± 11.87 ****	579.3 ± 11.94 ***	634.4 ± 20.87 **
		Axial FLAIR	622.4 ± 11.01 ****	585.7 ± 12.74 ***	636.7 ± 21.99 **
		Coronal T2	625.7 ± 9.35 ****	582.9 ± 12.10 **	629.4 ± 21.28 ns
Hippocampus	Reference Standard		**7.35 ± 0.20**	**6.50 ± 0.22**	**7.78 ± 0.26**
	Real sequence	3D FLAIR	7.02 ± 0.2 ns	6.23 ± 0.22 ns	7.52 ± 0.22 *
		Axial FLAIR	6.89 ± 0.19 ns	6.10 ± 0.22 *	7.30 ± 0.20 ns
		Coronal T2	6.37 ± 0.64 ns	6.7 ± 0.20 *	7.30 ± 0.25 ***
	Synthetic T1 generated from input	3D FLAIR	7.70 ± 0.19 ns	7.03 ± 0.15 *	8.14 ± 0.25 ****
		Axial FLAIR	7.70 ± 0.16 ns	7.03 ± 0.20 ns	7.81 ± 0.20 ns
		Coronal T2	7.92 ± 0.15 **	7.05 ± 0.18 *	8.03 ± 0.25 **

Supplementary Table 2: Global brain volumes and total hippocampus volumes resulting from volumetry with SynthSeg+ for Reference Standard real 3D T1, input sequences used for generation of synthetic sequences and synthetic T1 sequences from the different input sequences in cm^3 (Mean ± SEM). Stars indicate significance versus Reference Standard real 3D T1. Ns = not significant, * = p ≤ 0.05, ** = p ≤ 0.01, *** = p ≤ 0.001, **** = p ≤ 0.0001.

	AD	FTD	HC
**Whole brain**			
$sT1^{3DFLAIR}$ vs. r3DFLAIR	****	**	**
$sT1^{AxFLAIR}$ vs. rAxFLAIR	****	****	****
$sT1^{CorT2}$ vs. rCorT2	***	**	***
**White matter**			
$sT1^{3DFLAIR}$ vs. r3DFLAIR	ns	ns	**
$sT1^{AxFLAIR}$ vs. rAxFLAIR	ns	ns	ns
$sT1^{CorT2}$ vs. rCorT2	ns	ns	*
**Grey matter**			
$sT1^{3DFLAIR}$ vs. r3DFLAIR	****	****	***
$sT1^{AxFLAIR}$ vs. rAxFLAIR	****	****	****
$sT1^{CorT2}$ vs. rCorT2	****	****	****
**Hippocampus**			
$sT1^{3DFLAIR}$ vs. r3DFLAIR	**	**	****
$sT1^{AxFLAIR}$ vs. rAxFLAIR	***	***	**
$sT1^{CorT2}$ vs. rCorT2	***	****	****

Supplementary Table 3: Results from comparison of volumetric results using SynthSeg+ of deep learning-based synthetic 3D T1s versus direct volumetry of their respective input sequences. Ns = not significant, * = $p \leq 0.05$, ** = $p \leq 0.01$, *** = $p \leq 0.001$, **** = $p \leq 0.0001$.
